# 6,6′-Dimeth­oxy-2,2′-[*p*-phenyl­ene­bis(nitrilo­methyl­idyne)]diphenol chloro­form disolvate

**DOI:** 10.1107/S1600536809007302

**Published:** 2009-03-06

**Authors:** Mohammed H. Al-Douh, Hasnah Osman, Shafida Abd. Hamid, Reza Kia, Hoong-Kun Fun

**Affiliations:** a School of Chemical Sciences, Universiti Sains Malaysia, 11800 USM, Penang, Malaysia; b Kulliyyah of Science, International Islamic University Malaysia (IIUM), 25200 Kuantan, Pahang, Malaysia; c X-ray Crystallography Unit, School of Physics, Universiti Sains Malaysia, 11800 USM, Penang, Malaysia

## Abstract

The title compound, C_22_H_20_N_2_O_4_·2CHCl_3_, a new Schiff base compound, lies across a crystallographic inversion centre. An intra­molecular O—H⋯N hydrogen bond generates a six-membered ring, producing an *S*(6) ring motif. Inter­molecular bifurcated C—H⋯O hydrogen bonds involving the two O atoms of the Schiff base ligand and the H atom of the chloro­form solvent of crystallization, generate an *R* ^2^_1_(5) ring motif. The crystal structure is stabilized by inter­molecular C—H⋯π and π–π inter­actions [centroid to centroid distance = 3.6158 (10) Å]. In the crystal structure, mol­ecules are stacked down the *c* axis.

## Related literature

For hydrogen-bond motifs, see: Bernstein *et al.* (1995 ▶). For the synthesis and applications of Schiff bases see, for example: Salem & Amer (1995 ▶); Teoh *et al.* (1997 ▶); Viswanathamurthi *et al.* (1998 ▶); Cohen *et al.* (1964 ▶); Kabak *et al.* (2000 ▶); Parra *et al.* (2007 ▶); Al-Douh *et al.* (2006 ▶, 2007 ▶, 2008 ▶);  Liu *et al.* (2006 ▶); Shah *et al.* (2008 ▶). For the stability of the temperature controller used for the data collection, see: Cosier & Glazer (1986 ▶). For bond-length data, see: Allen *et al.* (1987 ▶).
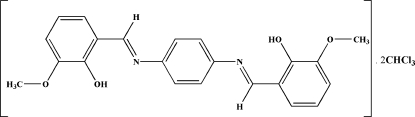


## Experimental

### 

#### Crystal data

C_22_H_20_N_2_O_4_·2CHCl_3_
*M* *_r_* = 615.14
Monoclinic, 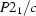

*a* = 10.4773 (2) Å
*b* = 21.3287 (5) Å
*c* = 6.2424 (2) Å
β = 105.669 (2)°
*V* = 1343.13 (6) Å^3^
*Z* = 2
Mo *K*α radiation
μ = 0.67 mm^−1^
*T* = 100 K
0.54 × 0.18 × 0.07 mm

#### Data collection

Bruker SMART APEXII CCD area-detector diffractometer
Absorption correction: multi-scan (**SADABS**; Bruker, 2005 ▶) *T*_min_ = 0.712, *T*_max_ = 0.958
10624 measured reflections
3069 independent reflections
2361 reflections with *I* > 2σ(*I*)
*R*_int_ = 0.033

#### Refinement

*R*[*F* ^2^ > 2σ(*F* ^2^)] = 0.036
*wR*(*F* ^2^) = 0.090
*S* = 1.03
3069 reflections
165 parameters
H-atom parameters constrained
Δρ_max_ = 0.35 e Å^−3^
Δρ_min_ = −0.25 e Å^−3^

### 

Data collection: *APEX2* (Bruker, 2005 ▶); cell refinement: *SAINT* (Bruker, 2005 ▶); data reduction: *SAINT*; program(s) used to solve structure: *SHELXTL* (Sheldrick, 2008 ▶); program(s) used to refine structure: *SHELXTL*; molecular graphics: *SHELXTL*; software used to prepare material for publication: *SHELXTL* and *PLATON* (Spek, 2009).

## Supplementary Material

Crystal structure: contains datablocks global, I. DOI: 10.1107/S1600536809007302/at2731sup1.cif

Structure factors: contains datablocks I. DOI: 10.1107/S1600536809007302/at2731Isup2.hkl

Additional supplementary materials:  crystallographic information; 3D view; checkCIF report

## Figures and Tables

**Table 1:** Hydrogen-bond geometry (Å, °)

*D*—H⋯*A*	*D*—H	H⋯*A*	*D*⋯*A*	*D*—H⋯*A*
O1—H1⋯N1	0.84	1.84	2.584 (2)	147
C12—H12*A*⋯O1^i^	1.00	2.21	3.120 (3)	150
C12—H12*A*⋯O2^i^	1.00	2.30	3.121 (3)	139
C3—H3*A*⋯*Cg*1^ii^	0.95	2.73	3.5221 (19)	142

Symmetry codes: (i) 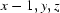; (ii) 
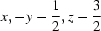
. *Cg*1 is the centroid of the C1–C6 benzene ring.

**Fig. 1.**
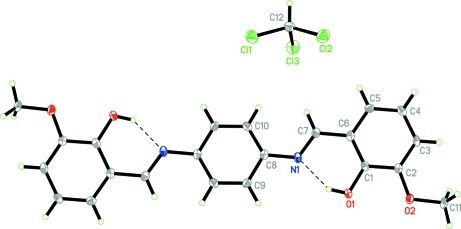
The molecular structure of the title compound, showing 50% probability displacement ellipsoids and the atomic numbering [symmetry code of unlabelled atoms -x + 1, -y, -z + 2]. Intramolecular H bonds are drawn as dashed lines.

**Fig. 2.**
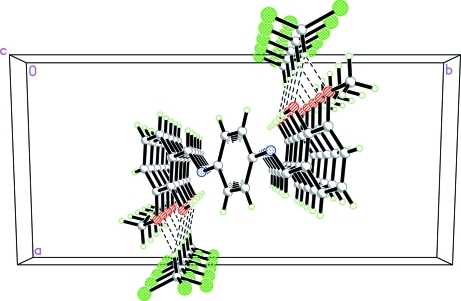
The crystal packing of the title compound, viewed down the c-axis showing stacking of molecules along the c-axis. Intermolecular hydrogen bonds are shown as dashed lines.

## supplementary crystallographic information

### Comment

Bis-Schiff bases are a class of important compounds used as pharmaceutical, medicinal and industrial materials. Schiff bases have also been used extensively in coordination and inorganic chemistry. Salem and Amer used H_2_O_2_ to study the kinetics of the oxidation of a manganese complex with bis-Schiff base of salicyldehyde (Salem & Amer, 1995). Many of these Schiff bases were found to form suitable inner coordination spheres between tin atom with O and N atoms as quadridentate chelates (Teoh *et al.*, 1997). Meanwhile, ruthenium complexes of bis-Schiff bases derived from *o*-vanillin and salicyldehyde were shown to exhibit dibasic tetradentate chelation (Viswanathamurthi *et al.*, 1998). The intramolecular hydrogen bonds formed between O and N atoms in Schiff bases are responsible for the formation of these metal complexes (Cohen *et al.*, 1964). Kabak *et al.* (2000) prepared the derivative of another isomer of the title compound and studied the photochromic conformational properties of this derivative, while Parra *et al.* (2007) examined the intercalation of another derivative of bis-Schiff bases with DNA by UV spectroscopy. Recently, we reported the crystal structure of the *meta*-isomer of the title compound (Al-Douh *et al.*, 2007), while the single-crystal of the second isomer in the *ortho*-position was obtained and the data are consistent with the reported structure (Liu *et al.*, 2006). The proton and carbon NMR spectroscopies of the title compound and its isomers were also studied (Al-Douh *et al.*, 2008). Our group has been actively involved in synthesizing bis-Schiff bases and investigating their DNA binding ability using spectroscopic techniques employing calf thymus DNA (Shah *et al.*, 2008). We have synthesized the third symmetric Schiff base by the condensation of *o*-vanillin with *p*-phenylenediamine and its *X*-ray crystal structure is presented here.

The title compound, (Fig. 1), lies across a crystallographic inversion centre [symmetry code of unlabelled atoms -*x* + 1, -*y*, -*z* + 2]. The bond lengths (Allen *et al.*, 1987) and angles are within normal ranges. An intramolecular O—H···N hydrogen bond generates a six-membered ring, producing *S*(6) ring motif (Bernstein *et al.*, 1995). Intermolecular bifurcated C—H···O hydrogen bonds involving the two oxygen atoms of the Schiff base ligand and the hydrogen atom of the chloroform solvent of crystallization generate a *R^2^_1_(5)* ring motif. There are short contacts [C1–C9 = 3.267 (3) and C2–C9 = 3.399 (3) Å] which are shorter than the sum of the van der Waals radius of carbon atom. The crystal structure is stabilized by intermolecular C—H···π interaction (*Cg*1 is the centroid of the C1–C6 benzene ring) (Table 1) and intermolecular π-π interaction [*Cg*1···*Cg*2^i, ii, iii, iv^ = 3.6158 (10) Å; symmetry codes: (i) *x*, 1/2 - *y*, 1/2 + *z* (ii) *x*, *y*, -1 + *z* (iii) 1 - *x*, -*y*, 1 - *z* (iv) *x*, *y*, 1 + *z, *(*Cg2 *is the centroid of the benzene ring in the middle of the main molecule)]. In the crystal structure molecule are stacked down the *c* axis (Fig. 2).

### Experimental

The synthetic method has been described earlier (Al-Douh *et al.*, 2006, 2007). Single crystals suitable for X-ray diffraction were obtained by slow evaporation of chloroform at room temperature.

### Refinement

H atoms of the hydroxy group was positioned by a freely rotating O—H bond and constrained with a fixed distance of 0.84 Å. The rest of the hydrogen atoms were positioned geometrically with a riding model approximation with C—H = 0.93–1.00 Å and *U*_iso_(H) = 1.2 or 1.5 (C & O).

### Crystal data

**Table:**

C_22_H_20_N_2_O_4_·2CHCl_3_	*F*(000) = 628
*M**_r_* = 615.14	*D*_x_ = 1.521 Mg m^−^^3^
Monoclinic, *P*2_1_/*c*	Mo *K*α radiation, λ = 0.71073 Å
Hall symbol: -P 2ybc	Cell parameters from 3653 reflections
*a* = 10.4773 (2) Å	θ = 2.8–29.6°
*b* = 21.3287 (5) Å	µ = 0.67 mm^−^^1^
*c* = 6.2424 (2) Å	*T* = 100 K
β = 105.669 (2)°	Plate, yellow
*V* = 1343.13 (6) Å^3^	0.54 × 0.18 × 0.07 mm
*Z* = 2	

### Data collection

**Table:**

Bruker SMART APEXII CCD area-detector diffractometer	3069 independent reflections
Radiation source: fine-focus sealed tube	2361 reflections with *I* > 2σ(*I*)
graphite	*R*_int_ = 0.033
φ and ω scans	θ_max_ = 27.5°, θ_min_ = 2.0°
Absorption correction: multi-scan (*SADABS*; Bruker, 2005)	*h* = −13→13
*T*_min_ = 0.712, *T*_max_ = 0.958	*k* = −27→27
10624 measured reflections	*l* = −8→8

### Refinement

**Table:**

Refinement on *F*^2^	Primary atom site location: structure-invariant direct methods
Least-squares matrix: full	Secondary atom site location: difference Fourier map
*R*[*F*^2^ > 2σ(*F*^2^)] = 0.036	Hydrogen site location: inferred from neighbouring sites
*wR*(*F*^2^) = 0.090	H-atom parameters constrained
*S* = 1.03	*w* = 1/[σ^2^(*F*_o_^2^) + (0.0405*P*)^2^ + 0.6965*P*] where *P* = (*F*_o_^2^ + 2*F*_c_^2^)/3
3069 reflections	(Δ/σ)_max_ < 0.001
165 parameters	Δρ_max_ = 0.35 e Å^−^^3^
0 restraints	Δρ_min_ = −0.24 e Å^−^^3^

### Special details

**Table:**

Experimental. The crystal was placed in the cold stream of an Oxford Cyrosystems Cobra open-flow nitrogen cryostat (Cosier & Glazer, 1986) operating at 100.0 (1) K.
Geometry. All e.s.d.'s (except the e.s.d. in the dihedral angle between two l.s. planes) are estimated using the full covariance matrix. The cell e.s.d.'s are taken into account individually in the estimation of e.s.d.'s in distances, angles and torsion angles; correlations between e.s.d.'s in cell parameters are only used when they are defined by crystal symmetry. An approximate (isotropic) treatment of cell e.s.d.'s is used for estimating e.s.d.'s involving l.s. planes.
Refinement. Refinement of *F*^2^ against ALL reflections. The weighted *R*-factor *wR* and goodness of fit *S* are based on *F*^2^, conventional *R*-factors *R* are based on *F*, with *F* set to zero for negative *F*^2^. The threshold expression of *F*^2^ > σ(*F*^2^) is used only for calculating *R*-factors(gt) *etc*. and is not relevant to the choice of reflections for refinement. *R*-factors based on *F*^2^ are statistically about twice as large as those based on *F*, and *R*- factors based on ALL data will be even larger.

### Fractional atomic coordinates and isotropic or equivalent isotropic displacement parameters (Å^2^)

**Table:**

	*x*	*y*	*z*	*U*_iso_*/*U*_eq_	
Cl1	0.05508 (5)	0.10427 (3)	0.75969 (9)	0.03053 (15)	
Cl2	0.09644 (5)	0.19372 (3)	0.43542 (10)	0.03061 (15)	
Cl3	0.06333 (6)	0.06178 (3)	0.32245 (10)	0.03145 (15)	
O1	0.71504 (12)	0.11003 (6)	0.4437 (2)	0.0159 (3)	
H1	0.6880	0.0909	0.5406	0.024*	
O2	0.76612 (13)	0.16921 (6)	0.1108 (2)	0.0167 (3)	
N1	0.54474 (15)	0.06871 (7)	0.6416 (3)	0.0131 (3)	
C1	0.61066 (18)	0.13631 (8)	0.2936 (3)	0.0126 (4)	
C2	0.63590 (18)	0.16924 (8)	0.1137 (3)	0.0125 (4)	
C3	0.53268 (19)	0.19797 (8)	−0.0409 (3)	0.0146 (4)	
H3A	0.5499	0.2209	−0.1606	0.017*	
C4	0.40308 (19)	0.19342 (8)	−0.0216 (3)	0.0159 (4)	
H4A	0.3325	0.2128	−0.1295	0.019*	
C5	0.37710 (18)	0.16110 (9)	0.1525 (3)	0.0153 (4)	
H5A	0.2888	0.1583	0.1643	0.018*	
C6	0.48096 (18)	0.13218 (8)	0.3132 (3)	0.0130 (4)	
C7	0.45272 (19)	0.09798 (8)	0.4963 (3)	0.0147 (4)	
H7A	0.3645	0.0971	0.5096	0.018*	
C8	0.51663 (18)	0.03447 (8)	0.8182 (3)	0.0126 (4)	
C9	0.62640 (18)	0.01408 (9)	0.9863 (3)	0.0153 (4)	
H9A	0.7133	0.0235	0.9766	0.018*	
C10	0.39007 (19)	0.01950 (9)	0.8339 (3)	0.0166 (4)	
H10A	0.3146	0.0325	0.7202	0.020*	
C11	0.7985 (2)	0.20505 (10)	−0.0612 (3)	0.0197 (4)	
H11A	0.8939	0.2020	−0.0459	0.029*	
H11B	0.7744	0.2490	−0.0487	0.029*	
H11C	0.7493	0.1887	−0.2067	0.029*	
C12	0.0159 (2)	0.12323 (10)	0.4736 (4)	0.0231 (5)	
H12A	−0.0821	0.1294	0.4173	0.028*	

### Atomic displacement parameters (Å^2^)

**Table:**

	*U*^11^	*U*^22^	*U*^33^	*U*^12^	*U*^13^	*U*^23^
Cl1	0.0318 (3)	0.0392 (3)	0.0202 (3)	0.0063 (2)	0.0064 (2)	0.0025 (2)
Cl2	0.0281 (3)	0.0258 (3)	0.0401 (4)	−0.0015 (2)	0.0128 (2)	0.0010 (2)
Cl3	0.0394 (3)	0.0294 (3)	0.0264 (3)	−0.0015 (2)	0.0102 (2)	−0.0048 (2)
O1	0.0158 (6)	0.0169 (7)	0.0145 (7)	0.0007 (5)	0.0034 (5)	0.0060 (6)
O2	0.0184 (7)	0.0165 (7)	0.0168 (7)	0.0003 (5)	0.0079 (6)	0.0046 (6)
N1	0.0192 (8)	0.0095 (7)	0.0115 (8)	−0.0006 (6)	0.0053 (6)	0.0004 (6)
C1	0.0184 (9)	0.0062 (8)	0.0120 (9)	0.0000 (7)	0.0023 (7)	−0.0009 (7)
C2	0.0165 (9)	0.0087 (8)	0.0127 (9)	−0.0017 (7)	0.0047 (7)	−0.0021 (7)
C3	0.0230 (10)	0.0084 (9)	0.0124 (10)	−0.0012 (7)	0.0050 (8)	0.0004 (7)
C4	0.0195 (9)	0.0115 (9)	0.0143 (10)	0.0017 (7)	0.0005 (8)	0.0010 (8)
C5	0.0144 (9)	0.0128 (9)	0.0189 (10)	0.0006 (7)	0.0047 (8)	0.0000 (8)
C6	0.0188 (9)	0.0084 (8)	0.0121 (9)	−0.0021 (7)	0.0050 (7)	−0.0023 (7)
C7	0.0170 (9)	0.0116 (9)	0.0167 (10)	−0.0024 (7)	0.0066 (8)	−0.0008 (7)
C8	0.0188 (9)	0.0080 (8)	0.0126 (9)	0.0003 (7)	0.0070 (7)	−0.0024 (7)
C9	0.0151 (9)	0.0138 (9)	0.0186 (10)	−0.0007 (7)	0.0072 (8)	0.0011 (8)
C10	0.0171 (9)	0.0148 (9)	0.0164 (10)	0.0021 (7)	0.0021 (8)	0.0024 (8)
C11	0.0220 (10)	0.0222 (11)	0.0179 (11)	−0.0021 (8)	0.0105 (8)	0.0048 (8)
C12	0.0190 (10)	0.0284 (12)	0.0214 (11)	0.0022 (8)	0.0049 (8)	0.0025 (9)

### Geometric parameters (Å, °)

**Table:**

Cl1—C12	1.768 (2)	C4—H4A	0.9500
Cl2—C12	1.771 (2)	C5—C6	1.408 (3)
Cl3—C12	1.763 (2)	C5—H5A	0.9500
O1—C1	1.354 (2)	C6—C7	1.452 (3)
O1—H1	0.8400	C7—H7A	0.9500
O2—C2	1.369 (2)	C8—C10	1.393 (3)
O2—C11	1.431 (2)	C8—C9	1.400 (3)
N1—C7	1.292 (2)	C9—C10^i^	1.381 (3)
N1—C8	1.418 (2)	C9—H9A	0.9500
C1—C6	1.400 (3)	C10—C9^i^	1.381 (3)
C1—C2	1.409 (3)	C10—H10A	0.9500
C2—C3	1.383 (3)	C11—H11A	0.9800
C3—C4	1.399 (3)	C11—H11B	0.9800
C3—H3A	0.9500	C11—H11C	0.9800
C4—C5	1.375 (3)	C12—H12A	1.0000
			
C1—O1—H1	109.5	C6—C7—H7A	119.2
C2—O2—C11	116.63 (14)	C10—C8—C9	118.75 (17)
C7—N1—C8	121.48 (16)	C10—C8—N1	125.04 (17)
O1—C1—C6	122.35 (17)	C9—C8—N1	116.21 (16)
O1—C1—C2	117.87 (16)	C10^i^—C9—C8	120.83 (18)
C6—C1—C2	119.78 (16)	C10^i^—C9—H9A	119.6
O2—C2—C3	125.84 (17)	C8—C9—H9A	119.6
O2—C2—C1	114.35 (15)	C9^i^—C10—C8	120.41 (17)
C3—C2—C1	119.81 (17)	C9^i^—C10—H10A	119.8
C2—C3—C4	120.26 (18)	C8—C10—H10A	119.8
C2—C3—H3A	119.9	O2—C11—H11A	109.5
C4—C3—H3A	119.9	O2—C11—H11B	109.5
C5—C4—C3	120.46 (17)	H11A—C11—H11B	109.5
C5—C4—H4A	119.8	O2—C11—H11C	109.5
C3—C4—H4A	119.8	H11A—C11—H11C	109.5
C4—C5—C6	120.21 (18)	H11B—C11—H11C	109.5
C4—C5—H5A	119.9	Cl3—C12—Cl1	110.39 (12)
C6—C5—H5A	119.9	Cl3—C12—Cl2	110.22 (12)
C1—C6—C5	119.47 (17)	Cl1—C12—Cl2	109.93 (12)
C1—C6—C7	120.62 (17)	Cl3—C12—H12A	108.7
C5—C6—C7	119.90 (17)	Cl1—C12—H12A	108.7
N1—C7—C6	121.54 (17)	Cl2—C12—H12A	108.7
N1—C7—H7A	119.2		
			
C11—O2—C2—C3	3.6 (3)	C2—C1—C6—C7	179.44 (17)
C11—O2—C2—C1	−176.48 (16)	C4—C5—C6—C1	−0.3 (3)
O1—C1—C2—O2	1.5 (2)	C4—C5—C6—C7	−179.84 (17)
C6—C1—C2—O2	−179.08 (16)	C8—N1—C7—C6	−178.93 (16)
O1—C1—C2—C3	−178.60 (16)	C1—C6—C7—N1	−2.4 (3)
C6—C1—C2—C3	0.9 (3)	C5—C6—C7—N1	177.12 (17)
O2—C2—C3—C4	178.67 (17)	C7—N1—C8—C10	11.6 (3)
C1—C2—C3—C4	−1.3 (3)	C7—N1—C8—C9	−169.16 (17)
C2—C3—C4—C5	0.9 (3)	C10—C8—C9—C10^i^	−0.9 (3)
C3—C4—C5—C6	−0.1 (3)	N1—C8—C9—C10^i^	179.83 (17)
O1—C1—C6—C5	179.37 (17)	C9—C8—C10—C9^i^	0.9 (3)
C2—C1—C6—C5	−0.1 (3)	N1—C8—C10—C9^i^	−179.90 (17)
O1—C1—C6—C7	−1.1 (3)		

Symmetry codes: (i) −*x*+1, −*y*, −*z*+2.

### Hydrogen-bond geometry (Å, °)

**Table:**

*D*—H···*A*	*D*—H	H···*A*	*D*···*A*	*D*—H···*A*
O1—H1···N1	0.84	1.84	2.584 (2)	147
C12—H12A···O1^ii^	1.00	2.21	3.120 (3)	150
C12—H12A···O2^ii^	1.00	2.30	3.121 (3)	139
C3—H3A···Cg1^iii^	0.95	2.73	3.5221 (19)	142

Symmetry codes: (ii) *x*−1, *y*, *z*; (iii) *x*, −*y*−1/2, *z*−3/2.
